# Serum Neurofilament Light Predicts 6-Month Mental Health Outcomes in a Cohort of Patients With Acute Ischemic Stroke

**DOI:** 10.3389/fpsyt.2021.764656

**Published:** 2022-02-07

**Authors:** Duo-Zi Wang, Fu-Qiang Guo, Lei Guo, Shu Yang, Neng-Wei Yu, Jian Wang, Jian-Hong Wang

**Affiliations:** ^1^Department of Neurology, The Affiliated Hospital of University of Electronic Science and Technology, Sichuan Provincial People's Hospital, Chengdu, China; ^2^Department of Neurology, Ya'an People's Hospital, Ya'an, China

**Keywords:** post-stroke, neurofilament light, depression, anxiety, insomnia

## Abstract

**Background:**

Mental health problems after acute ischemic stroke (AIS) have caused wide public concerns, and the study on early identification of these disorders is still an open issue. This study aims to investigate the predictive effect of circulating neurofilament light (NfL) on long-term mental health status of AIS patients.

**Methods:**

This study collected demographic information and mental health measurements from 304 AIS patients from May 1, 2016 to Dec 31, 2019. Baseline serum neurofilament light (NfL) was determined within 2 h since patient admission. Six months after AIS onset, the degree of symptoms of depression, anxiety, and insomnia was assessed by the Chinese versions of the 9-item Patient Health Questionnaire (PHQ-9), the 7-item Generalized Anxiety Disorder scale (GAD-7), the 7-item Insomnia Severity Index (ISI), respectively. Subjects were divided into the high NfL group and the low NfL group. Multivariate logistic regression analysis was performed to identify factors associated with these mental health problems.

**Results:**

The high NfL group had significantly higher PHQ-9, GAD-7, and ISI scores than the low NfL group. The prediction of serum NfL for major depression generated a sensitivity of 70.27%, a specificity of 67.79% and an AUC of 0.694. The prediction of serum NfL for anxiety generated a sensitivity of 69.23%, a specificity of 64.02%, and an AUC of 0.683. The prediction of serum NfL for insomnia generated a sensitivity of 75.00%, a specificity of 66.43% and an AUC of 0.723. Higher serum NfL was a risk factor of post-AIS depression [ORs (95% CI): 4.427 (1.918, 10.217)], anxiety [ORs (95% CI): 3.063 (1.939, 6.692)], and insomnia [ORs (95% CI): 4.200 (1.526, 11.562)].

**Conclusions:**

These findings imply that circulating NfL might be a potential biomarker of long-term mental health problems after AIS.

## Introduction

Acute ischemic stroke (AIS) is among the leading causes of death and disability worldwide (1). Except for neurological deficits, AIS patients also experience a variety of mental health problems, such as anxiety (2), depression (3), and insomnia (4) during the rehabilitation of the disease. Although these post-AIS consequences do not directly cause death or disability, they are closely related to the quality of life after stroke. Therefore, early identification of patients with risk of developing neuropsychological disorders is of significance for timely intervention to improve the mental health outcomes. Recent studies have identified a panel of blood-based biomarkers that is associated with stroke severity and prognosis (5). However, few convenient and effective biomarkers are available to evaluate the risk of neuropsychological problems after stroke.

Neurofilaments, including neurofilament light (NfL), neurofilament medium (NfM), and neurofilament heavy (NfH) are components of the neuronal cytoskeleton. Together with NfH and NfM, NfL represents one of the scaffolding proteins of the neuronal cytoskeleton and is released into the extracellular space following neuronal damage (6). NfL is increased in multiple neurological diseases, such as Alzheimer's disease (7), Parkinson's disease (8), and multiple sclerosis (9). It is also a well-validated prognostic biomarker of functional outcomes of AIS (10). But it is not clear yet whether NfL could predict mental health outcomes of this disease. This study aims to investigate the association between circulating NfL and mental health outcomes of AIS, including major depression, anxiety, and insomnia.

## Subjects and Methods

### Patients

This study used the same cohort of patients in our previous study (11). Briefly, inpatients with AIS from the Department of Neurology, Sichuan Provincial People's Hospital and Ya'an People's Hospital during May 1, 2016 and Dec 31, 2019, were screened for eligibility for this study. Patients were excluded if they have one of the following conditions: (1) Have previously diagnosed depression, anxiety, and insomnia before AIS onset; (2) have other psychological disorders, such as schizophrenia and bipolar disorders et al. before AIS onset; (3) Cannot complete psychological tests due to hearing, language, or communicating disabilities; (4) Other severe neurological diseases which may affect circulating NfL levels, such as Parkinson's disease, Alzheimer's disease, and traumatic brain injury; (5) Refused to participate in this study. Written informed consent for participation was obtained from patients or their legal relatives. This study conformed with the principles of the Declaration of Helsinki and was approved by the investigational review board of the Sichuan Provincial People's Hospital.

### Clinical Assessment and Data Collection

The demographic information, including age, sex, education level, body mass index (BMI), smoking history, medical history, including oral anticoagulion or antiplatelet drug use, comorbidities such as hypertension, diabetes mellitus, hypercholesteremia, and arial fibrillation were collected from the medical records. AIS was diagnosed according to the World Health Organization (WHO) Multinational Monitoring of Trends and Determinants in Cardiovascular Disease (WHO-MONICA) criteria and was verified by magnetic resonance imaging (MRI) performed within 24 h since symptom onset. The neurological deficits of patients were examined with the National Institutes of Health Stroke Scale (NIHSS) upon admission (12), performed by a certified stroke neurologist. AIS subtype was determined with the TOAST criteria.

### Assessment of Mental Health Outcomes

We focused on symptoms of depression, anxiety, and insomnia for all participants 6 months after AIS onset, using Chinese versions of validated measurement tools. Accordingly, the 9-item Patient Health Questionnaire (PHQ-9; range, 0–27) (13), the 7-item Generalized Anxiety Disorder (GAD-7) scale (range, 0–21) (14), the 7-item Insomnia Severity Index (ISI; range, 0–28) (15), were used to assess the severity of symptoms of depression, anxiety, and insomnia, respectively. The total scores of these measurement tools were interpreted as follows: PHQ-9, normal (0–4), mild (5–9), moderate (10–14), and severe (15–27) depression; GAD-7, normal (0–4), mild (5–9), moderate (10–14), and severe (15–21) anxiety; ISI, normal (0–7), subthreshold (8–14), moderate (15–21), and severe (22–28) insomnia. These categories were based on cutoff values established in the literature (13–15). The cutoff value for detecting symptoms of major depression, anxiety, and insomnia distress were 10, 7, and 15, respectively. Participants with scores above the cutoff threshold were characterized as having major depression, anxiety and insomnia, respectively.

### NfL Concentration Determination

Blood was sampled within 2 h since admission, and serum was separated within 30 min after sampling and stored at −80°C until further analysis. Serum NfL was determined using the single-molecule (Simoa) array according to manufacturer's instructions (16). Each test was done in duplicates and the means of each test were used for statistical analysis. Monoclonal antibodies and purified bovine NFL were used as calibrators.

### Statistical Analysis

Continuous variables were tested for normality, and if they were normally distributed, an independent *t*-test was used, but if they were not normally distributed, a Mann-Whitney *U*-test was used. For categorical data, two-sample tests of proportions were used to compare proportions. Logistical regression models were utilized to investigate the association between serum NfL concentrations at baseline and mental health outcomes. We first fitted univariate models with a single candidate variable at one time. The potential risk factors as determined by a *p*-value < 0.2 were included in the final multivariate regression models. The receiver operating characteristic (ROC) curve analysis were utilized to test the predictive effects of baseline serum NfL on mental health outcomes at follow-up. Optimal sensitivity and specificity were determined *via* a non-parametric approach. The Youden index was calculated for the cutoff value to determine the cutoff value that maximized the discriminating power of the test. Statistical analyses were conducted using SPSS statistical package version 24 (IBM SPSS Statistics for Windows, Armonk, NY, USA) and a *p*-value < 0.05 was regarded as statistically significant.

## Results

### Demographic Characteristics of Subjects

We categorized the patients into two groups according to serum NfL concentrations, the high NfL group and the low NfL group. There was no significant difference in the mean age, median BMI, the frequency of family history of stroke, frequencies of antiplatelet drug use and anticoagulation drug use between the high NfL and low NfL group. Frequencies of hypertension, diabetes mellitus, hypercholesteremia and arial fibrillation between the high NfL and low NfL group were also not significantly different between groups. No significant difference was not observed in the distribution of infarction region and stroke etiology between the high and low NfL group. No significant difference was also not observed in the incidences of hemorrhagic transformation and recurrent AIS during follow-up between groups. The high NfL group had significantly higher incidences of PSD, PSA, and PSI than the low NfL group during follow-up (Table 1).

**Table 1:** Demographic information of subjects.

**Variables**	**Low NfL group (*n* = 152)**	**High NfL group (*n* = 152)**	***P*** **-value**
Age, mean (SD)	64.81 (9.25)	65.02 (9.25)	0.843^*a*^
Female, No. (%)	88 (57.89)	86 (56.58)	0.908^*b*^
BMI, median (IQR)	24.51 (23.08–25.59)	24.24 (23.08–25.54)	0.483^*c*^
Smoking history, No. (%)	11 (7.24)	17 (11.18)	0.321^*b*^
Antiplatelet drug use, No. (%)	20 (13.16)	20 (13.16)	1.000^*b*^
Antithrombotic drug use, No. (%)	5 (3.29)	14 (9.21)	0.055^*b*^
Family history of stroke, No. (%)	7 (4.61)	11 (7.24)	0.467^*b*^
**Comorbidities**			
Hypertension, No. (%)	49 (32.24)	55 (36.18)	0.546^*b*^
Diabetes Mellitus, No. (%)	28 (18.42)	21 (13.82)	0.349^*b*^
Hypercholesteremia, No. (%)	14 (9.21)	14 (9.21)	1.000^*b*^
Arial fibrillation, No. (%)	5 (3.29)	14 (9.21)	0.055^*b*^
Post stoke anxiety, No. (%)	17 (11.18)	48 (31.58)	<0.001^*b*^
Post stroke depression, No. (%)	8 (5.26)	29 (19.08)	<0.001^*b*^
Post stroke insomnia, No. (%)	5 (3.29)	19 (12.50)	<0.001^*b*^
DWI hyperintensity volume, ml (SD)	24.37 (1.52)	24.26 (1.50)	0.544^*b*^
Infarction region^*^			
Cerebral lobe, No. (%)	29 (19.08)	23 (15.13)	0.447^*b*^
Cerebral white matter, No. (%)	26 (17.11)	20 (13.16)	0.424^*b*^
Striatocapsule, No. (%)	103 (67.76)	112 (73.68)	0.313^*b*^
Thalamus, No. (%)	4 (2.63)	8 (5.26)	0.378^*b*^
Cerebellum, No. (%)	4 (2.63)	6 (3.95)	0.750^*b*^
**Stroke etiology**			
Atherothrombotic, No. (%)	132 (86.84)	125 (82.24)	0.341^*b*^
Cardioembolic, No. (%)	5 (3.29)	14 (9.21)	0.055^*b*^
Lacunar, No. (%)	9 (5.92)	8 (5.26)	1.000^*b*^
Unknown, No. (%)	6 (3.95)	5 (3.29)	1.000^*b*^
**Complication**			
Hemorrhagic effect, No. (%)	4 (2.63)	6 (3.95)	0.750^*b*^
Recurrent AIS, No. (%)	3 (1.97)	2 (1.32)	1.000^*b*^
Post-stroke depression, No. (%)	8 (5.26)	29 (19.08)	<0.001^*b*^
Post-stroke anxiety, No. (%)	17 (11.18)	48 (31.58)	<0.001^*b*^
Post-stroke insomnia, No. (%)	5 (3.29)	19 (12.50)	0.005^*b*^

*IQR, Inter-Quartile Range; BMI, Body Mass Index; NIHSS, National Institutes of Health Stroke Scale*.

* *It is notable that the infarctions may involve multiple brain regions*.

a *Unpaired t-test*.

b *Pearson χ^2^-test*.

c *Mann-Whitney U-test*.

### Mental Health Outcomes of AIS Patients and Their Associations With Serum NfL Levels

**Figure 1:**
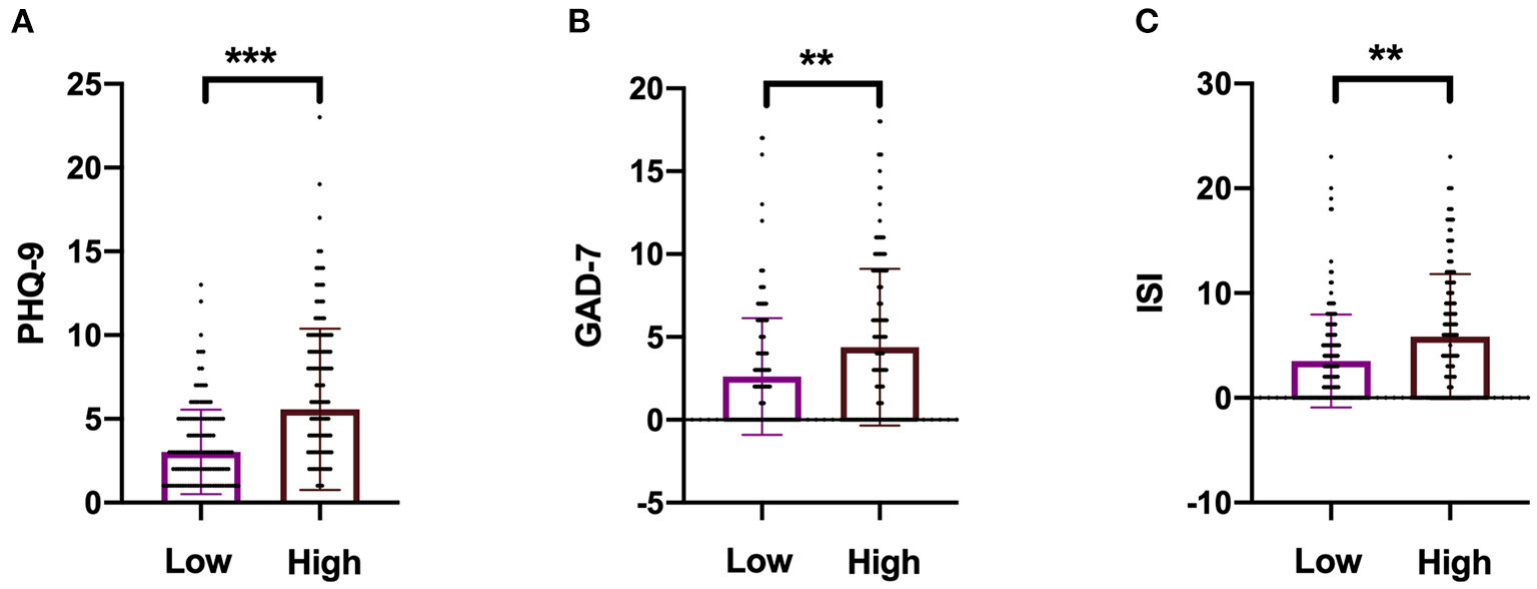
Comparison of the severity of depression, anxiety, and insomnia between high and low NfL group. **(A)** Comparison of PHQ-9 score between high and low NfL group. **(B)** Comparison of GAD-7 score between high and low NfL group. **(C)** Comparison of ISI score between high and low NfL group. PHQ-9, GAD-7, and ISI scales were used to determine the severity of depression, anxiety, and insomnia of subjects. Unimpaired *t*-test. **p < 0.01, ***p < 0.001.

**Figure 2:**
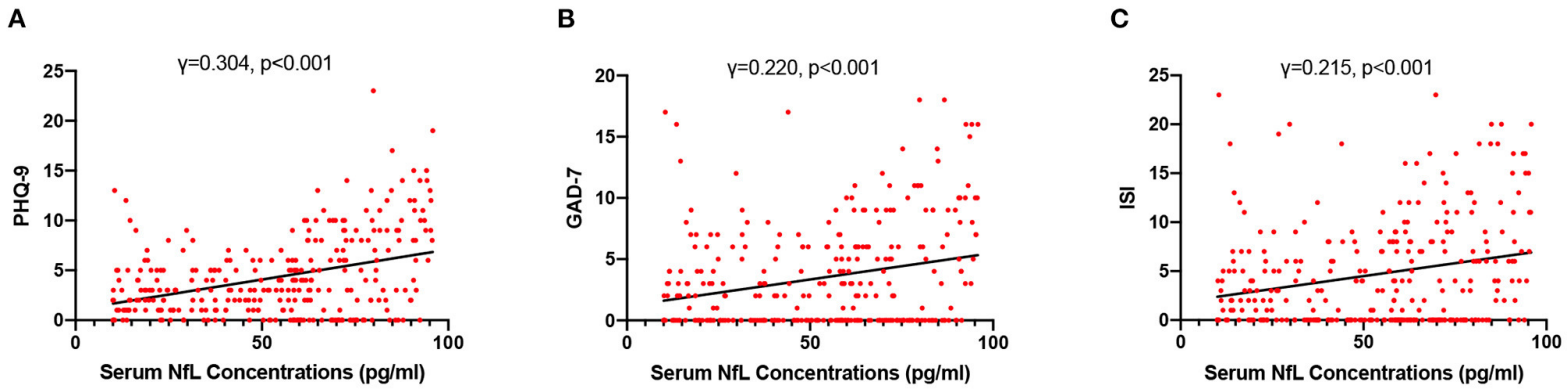
Association between serum NfL levels and post-AIS mental health outcomes. **(A)** Association between serum NfL concentrations and PHQ-9 score. **(B)** Association between serum NfL concentrations and GDA-7 score. **(C)** Association between serum NfL concentrations and ISS score. Spearman correlation analysis.

**Figure 3:**
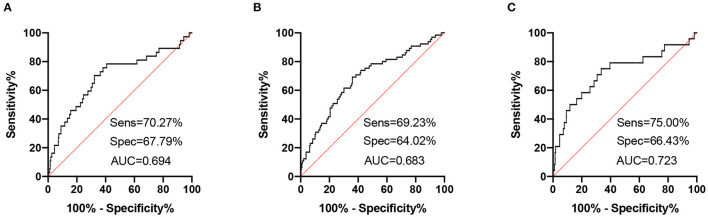
Receiver operating characteristic (ROC) curves of serum NfL for post-AIS mental health outcomes. **(A)** ROC curve of serum NfL and PHQ-9 score. **(B)** ROC curve of serum NfL and GAD-7 score. **(C)** ROC curve of serum NfL and ISI score.

High NfL group had significantly higher PHQ-9, GAD-7, and ISI scores than low NfL group (Figure 1). Serum NfL concentrations were positively associated with PHQ-9, GAD-7, and ISI scores (Figure 2). Furthermore, serum NfL predicted major depression with a sensitivity of 70.27%, a specificity of 67.79% and an AUC of 0.694 (Figure 3A). Serum NfL predicted anxiety with a sensitivity of 69.23%, a specificity of 64.02% and an AUC of 0.683 (Figure 3B). Serum NfL predicted insomnia with a sensitivity of 75.00%, a specificity of 66.43% and an AUC of 0.723 (Figure 3C).

### Risk Factors of Mental Health Problems

We utilized three logistic regression models to investigate risk factors of major depression, anxiety and insomnia. In univariate analyses, family history of stroke [ORs (95% CI): 3.053 (1.021, 9.125)], DWI hyperintensity volume [ORs (95% CI): 1.037 (0.998, 1.077)], cerebral lobe infarction [ORs (95% CI): 1.984 (0.894, 4.402)], and high NfL level [ORs (95% CI): 4.244 (1.871, 9.625)] were found to be potential risk factors of major depression. Male sex was found to be a protective factor against major depression [ORs (95% CI): 0.526 (0.262, 1.053)]. However, only high NfL level remained to be a significant risk factor of major depression [ORs (95% CI): 4.427 (1.918, 10.217)] in the final multivariate model (Table 2).

**Table 2:** A logistic regression model to evaluate the association between serum NfL and post-stroke major depression.

**Variables**	**Univariate ORs (95%CI)**	***P*** **-value**	**Multivariate ORs (95%CI)**	***P*** **-value**
Age, year	1.004 (0.968, 1.042)	0.817		
Sex, male	**0.526 (0.262, 1.053)**	**0.070**	0.613 (0.287, 1.312)	0.208
BMI, kg/m^2^	0.963 (0.767, 1.209)	0.746		
Smoking history, vs. no	1.227 (0.401, 3.758)	0.720		
Antiplatelet drug use, vs. no	1.655 (0.673, 4.069)	0.273		
Family history of stroke, vs. no	**3.053 (1.021, 9.125)**	**0.046**	2.698 (0.851, 8.557)	0.092
**Co-existing disorders**				
Hypertension, vs. no	1.197 (0.588, 2.438)	0.620		
Diabetes Mellitus, vs. no	0.792 (0.292, 2.145)	0.646		
Hypercholesteremia, vs. no	0.530 (0.120, 2.330)	0.400		
Arial fibrillation, vs. no	0.840 (0.186, 3.794)	0.821		
DWI hyperintensity volume, ml	**1.037 (0.998, 1.077)**	**0.066**	1.031 (0.975, 1.091)	0.281
Stroke etiology	0.913 (0.551, 1.513)	0.725		
**Infarction region**				
Cerebral lobe infarction, vs. no	**1.984 (0.894, 4.402)**	**0.092**	1.146 (0.326, 4.025)	0.832
Cerebral white matter infarction, vs. no	1.364 (0.560, 3.322)	0.494		
Striatocapsule infarction, vs. no	0.562 (0.277, 1.143)	0.111		
Thalamus infarction, vs. no	2.259 (0.653, 9.803)	0.179		
Cerebellum infarction, vs. no	0.796 (0.098, 6.472)	0.831		
Delirium, vs. no	1.033 (0.225, 4.737)	0.967		
Hemorrhagic transformation, vs. no	0.000 (0.000, ~)	0.999		
Recurrent stroke, vs. no	1.826 (0.199, 16.796)	0.595		
High NfL level, vs. low NfL level	**4.244 (1.871, 9.625)**	**0.001**	**4.427 (1.918, 10.217)**	**<0.001**

*In univariate analyses, variables with a p-value < 0.100 were included in the multivariate analysis. The bold caption represented meaningful association*.

In univariate analyses, family history of stroke [ORs (95% CI): 2.502 (0.929, 6.736)], thalamus infarction [ORs (95% CI): 2.762 (0.847, 9.008)], and high NfL level [ORs (95% CI): 3.665 (1.993, 6.742)] were found to be potential risk factors of anxiety. Male sex was found to be a protective factor against anxiety [ORs (95% CI): 0.522 (0.300, 0.908)]. However, only high NfL level [ORs (95% CI): 3.063 (1.939, 6.692)] remained to be a significant risk factor of anxiety and male sex [ORs (95% CI): 0.514 (0.288, 0.917)] remained to be a protective factor against that in the final multivariate model (Supplementary Table 1).

In the analysis of risk factors of insomnia, only high NfL level [ORs (95% CI): 4.200 (1.526, 11.562)] was found to be a risk factor in both univariate and multivariate analyses. Collectively, these findings indicate that high serum NfL level might be a potential risk factor of major depression, anxiety, and insomnia (Supplementary Table 2).

## Discussion

In the present study, we investigated the associations between circulating NfL levels and mental health outcomes of AIS. We found that the incidences of mental health disorders after AIS, including anxiety, depression and insomnia, were significantly higher in the high NfL group in comparison with the low NfL group. ROC analyses found that serum NfL had a relatively high accuracy of predicting the occurrence of major depression, anxiety and insomnia. Furthermore, regression models identified high NfL level as a risk factor of these mental health disorders.

Post-stroke mental health disorders, including PSD (17), PSA (18), and PSI (19), are harmful to the quality of life in patients with AIS. Furthermore, these neuropsychological consequences of AIS have adverse effects on functional improvement of AIS. Therefore, early identification of patients at risk of developing these disorders is essential for timely intervention to achieve better prognosis. However, currently no reliable biomarker is available to predict the mental health outcomes of AIS.

Mounting evidence has demonstrated the association between circulating NfL levels and functional outcomes of AIS (10, 20). NfL is also suggested to be a reliable biomarker predicting post-stroke cognitive impairment (11, 21, 22). Mental health disorders, including depression, anxiety and insomnia, are commonly observed during the rehabilitation of AIS (23–25). The prevalence of PSD, PSA, and PSI was 12.17, 21.38, and 7.89%, respectively in this study, which is comparable to other reports in the Chinese population (2, 26). Therefore, in this study, we investigated the predictive value of circulating NfL for the 6-month neuropsychological outcomes of AIS.

We categorized the AIS patients into two subgroups according to serum NfL level, and it is interesting to see that high NfL group had substantially higher incidences of PSD, PSA, and PSI than low NfL group. Circulating NfL could predict post-AIS mental health disorders with a relatively high accuracy. In a recent study, it is demonstrated that elevated level of circulating NfL is associated with an increased risk of depression 3 months after stroke onset (27), which is consistent with our present findings. Although patients with anxiety (28) and insomnia (29) are found to have increased NfL levels, the association of circulating NfL with these disorders in AIS has not been reported yet.

The mechanisms underlying the associations between NfL and post-stroke mental health disorders could be multifactual and have not been thoroughly illustrated. NfL is also found to be increased in other psychiatric disorders, such as bipolar disorders (30), chronic insomnia disorder (29), and depression not due to stroke (31). These findings suggest that mental health disorders might contribute to neuronal damage, thus promoting the increase of NfL. In return, it is not clear why AIS patients with high circulating NfL levels are more prone to develop these mental illnesses. But we could speculate that mental health disorders are associated with the severity of neuroaxonal damage, as reflected by NfL levels. We propose that the disruption of the equilibrium of neurotransmitters induced by ischemic attack may induce neuroaxonal damage and promote might contribute to post-AIS mental health disorders (32).

This study has several limitations. First, this is a simple correlation analysis with a relatively small sample size, further large-scale investigations are needed to confirm the present findings. Second, only baseline NfL levels were determined, thus the association between the dynamic change of NfL and neuropsychological outcomes of AIS patients has not been illustrated. But in conclusion, this study found NfL as a potential biomarker of post-stroke mental health disorder, including depression, anxiety and insomnia. Furthermore, the median age of participants in this study is more than 60, thus it is unclear whether NfL could predict long-term mental health disorders in young AIS patients. Patients with increased NfL levels should be intensively monitored for delayed neuropsychological disorders.

## Data Availability Statement

The original contributions presented in the study are included in the article/Supplementary Material, further inquiries can be directed to the corresponding author.

## Ethics Statement

The studies involving human participants were reviewed and approved by Investigational Review Board of the Sichuan Provincial People's Hospital. The patients/participants provided their written informed consent to participate in this study.

## Author Contributions

J-HW designed the study and drafted the manuscript. D-ZW and F-QG collected the samples and patients' information. D-ZW, SY, and N-WY participated in the determination of NfL. D-ZW and N-WY conducted the statistical analysis. GL and WJ contributed to the revision of the manuscript. All authors contributed to the article and approved the submitted version.

## Conflict of Interest

The authors declare that the research was conducted in the absence of any commercial or financial relationships that could be construed as a potential conflict of interest.

## Publisher's Note

All claims expressed in this article are solely those of the authors and do not necessarily represent those of their affiliated organizations, or those of the publisher, the editors and the reviewers. Any product that may be evaluated in this article, or claim that may be made by its manufacturer, is not guaranteed or endorsed by the publisher.
