# Supplementary material for: Serum Neurofilament Light Predicts 6-Month Mental Health Outcomes in a Cohort of Patients With Acute Ischemic Stroke
Source: Front Psychiatry. 2022 Feb 7;12:764656. doi: 10.3389/fpsyt.2021.764656 (PMC8859250; doi:10.3389/fpsyt.2021.764656)
Supplement: Supplementary file 1 [file Data_Sheet_1.docx]

**Supplementary Table 1. A logistic regression model to evaluate the association between serum NfL and post-stroke anxiety.**

| Variables | Univariate  ORs (95%CI) | *P* value | Multivariate  ORs (95%CI) | *P* value |
| --- | --- | --- | --- | --- |
| Age, year | 0.998 (0.968, 1.028) | 0.874 |  |  |
| Sex, male | **0.522 (0.300, 0.908)** | **0.021** | **0.514 (0.288, 0.917)** | **0.024** |
| BMI, kg/m^2^ | 0.913 (0.761, 1.095) | 0.325 |  |  |
| Smoking history, vs. no | 0.448 (0.196, 1.025) | 0.057 |  |  |
| Antiplatelet drug use, vs. no | 1.706 (0.814, 3.577) | 0.157 |  |  |
| Family history of stroke, vs. no | **2.502 (0.929, 6.736)** | **0.070** | 2.137 (0.744, 6.136) | 0.158 |
| Co-existing disorders |  |  |  |  |
| Hypertension, vs. no | 1.163 (0.657, 2.059) | 0.603 |  |  |
| Diabetes Mellitus, vs. no | 1.475 (0.654, 3.326) | 0.348 |  |  |
| Hypercholesteremia, vs. no | 0.587 (0.196, 1.758) | 0.341 |  |  |
| Arial fibrillation, vs. no | 0.979 (0.314, 3.058) | 0.971 |  |  |
| DWI hyperintensity volume, ml | 1.018 (0.987, 1.049) | 0.257 |  |  |
| Stroke etiology | 1.059 (0.735, 1.526) | 0.757 |  |  |
| Infarction region |  |  |  |  |
| Cerebral lobe infarction, vs. no | 1.638 (0.834, 3.217) | 0.152 |  |  |
| Cerebral white matter infarction, vs. no | 1.561 (0.767, 3.175) | 0.219 |  |  |
| Striatocapsule infarction, vs. no | 0.638 (0.358, 1.138) | 0.128 |  |  |
| Thalamus infarction, vs. no | **2.762 (0.847, 9.008)** | **0.092** | 2.469 (0.703, 8.673) | 0.158 |
| Cerebellum infarction, vs. no | 0.399 (0.050, 3.211) | 0.388 |  |  |
| Delirium, vs. no | 1.240 (0.386, 3.982) | 0.717 |  |  |
| Hemorrhagic transformation, vs. no | 0.000 (0.000, ~) | 0.999 |  |  |
| Recurrent stroke, vs. no | 0.918 (0.101, 8.357) | 0.939 |  |  |
| High NfL level, vs. low NfL level | **3.665 (1.993, 6.742)** | **<0.001** | **3.063 (1.939, 6.692)** | **<0.001** |

In univariate analyses, variables with a p value less than 0.100 were included in the multivariate analysis.

**Supplementary Table 2. A logistic regression model to evaluate the association between serum NfL and post-stroke insomnia.**

| Variables | Univariate  ORs (95%CI) | *P* value | Multivariate  ORs (95%CI) | *P* value |
| --- | --- | --- | --- | --- |
| Age, year | 1.007 (0.963, 1.053) | 0.763 |  |  |
| Sex, male | 0.607 (0.263, 1.403) | 0.243 |  |  |
| BMI, kg/m^2^ | 0.979 (0.743, 1.291) | 0.883 |  |  |
| Smoking history, vs. no | 2.133 (0.674, 6.752) | 0.197 |  |  |
| Antiplatelet drug use, vs. no | 0.938 (0.267, 3.302) | 0.921 |  |  |
| Family history of stroke, vs. no | 2.524 (0.676, 9.418) | 0.168 |  |  |
| Co-existing disorders |  |  |  |  |
| Hypertension, vs. no | 0.958 (0.396, 2.319) | 0.925 |  |  |
| Diabetes Mellitus, vs. no | 1.376 (0.394, 4.805) | 0.617 |  |  |
| Hypercholesteremia, vs. no | 0.888 (0.198, 3.991) | 0.877 |  |  |
| Arial fibrillation, vs. no | 1.406 (0.305, 6.485) | 0.662 |  |  |
| DWI hyperintensity volume, ml | 1.025 (0.979, 1.074) | 0.284 |  |  |
| Stroke etiology | 0.738 (0.351, 1.552) | 0.424 |  |  |
| Infarction region |  |  |  |  |
| Cerebral lobe infarction, vs. no | 1.696 (0.639, 4.502) | 0.289 |  |  |
| Cerebral white matter infarction, vs. no | 1.133 (0.369, 3.482) | 0.827 |  |  |
| Striatocapsule infarction, vs. no | 0.667 (0.280, 1.585) | 0.359 |  |  |
| Thalamus infarction, vs. no | 1.063 (0.131, 8.604) | 0.954 |  |  |
| Cerebellum infarction, vs. no | 1.309 (0.159, 10.792) | 0.802 |  |  |
| Delirium, vs. no | 1.727 (0.369, 8.090) | 0.488 |  |  |
| Hemorrhagic transformation, vs. no | 0.000 (0.000, ~) | 0.999 |  |  |
| Recurrent stroke, vs. no | 3.000 (0.322, 27.961) | 0.335 |  |  |
| High NfL level, vs. low NfL level | **4.200 (1.526, 11.562)** | **0.005** | **4.200 (1.526, 11.562)** | **0.005** |

In univariate analyses, variables with a p value less than 0.100 were included in the multivariate analysis.
